# The London Hospital's Arrangements

**Published:** 1914-08-22

**Authors:** 


### The London Hospitals' Arrangements.

### GUY'S HOSPITAL.

*(From our Special Correspondent.)*

Sir Cooper Perry, medical superintendent of Guy's, has addressed letters to the War Office, in the name of the governors and staff, offering help in this national emergency. The scheme includes the novel proposition of providing 150 additional beds in wooden huts in the "Park," this extension to be fully equipped and worked as part of the hospital by the medical and surgical staff, house officers, students, sisters, and nurses.

Another most valuable offer is that, noted last week, of the Guy's Dental School. Fifty dental chairs, available for use for six hours each day, to be reserved for the use of recruits rejected on the ground of defective teeth. The War Office is only to be charged the actual cost price of all material used in conservation treatment.

The medical school appointments, as in other hospitals, have received revision, and many familiar faces have disappeared in answer to the call of the Services. Those remaining are undertaking extra work to fill up the gaps, and perhaps, most difficult of all, learning.

The registrars are holding classes for medical students in ambulance work and the sisters in nursing services, which will render men efficient dressers and orderlies if called upon by the War Office. The ever-useful "Park" is now the scene of the daily 9.25 a.m. squad and stretcher drill of the University of London Officers' Training Corps, Medical Unit, B Section, under Captain T. B. Layton. Saturday 2 p.m. field work includes, we hear, the digging of pits for camp fires. Orders posted in the Colonnade and the number of men in uniform about the grounds have considerably changed the appearance of the hospital. Many of the senior staff of Guy's and St. Thomas's will be on duty at the Base Hospital, Chelsea, No. 2 Territorial.

### METROPOLITAN HOSPITAL.

*(From our Special Correspondent.)*

Twenty-four beds have been offered to the Government, with the promise of more if and when occasion requires. The committee feel, however, that in this poor district, especially if the German Hospital reduces its work, special provision must be made for the women and children. The visiting staff are all present to attend to the patients; but only three residents are left at the present time, and it is impossible to get any more.

The out-patient department has had to be closed for a fortnight for very necessary painting.

Several contractors—meat, bread, tea, and groceries—have given notice that they have temporarily to increase their prices. On the representations of the committee, however, they are now abiding by their contracts.

### ST. GEORGE'S HOSPITAL.

*(From our Special Correspondent.)*

The House Committee of St. George's Hospital have placed at the disposal of the Admiralty and War Office 100 beds and the hospital's convalescent branch at Wimbledon. The whole of the medical staff are co-operating so as to contribute to the Services, and to keep up the efficiency of the hospital. Of the visiting staff, three of the physicians and two of the surgeons have been called away on active duty; and the remainder of the senior staff are gazetted for service at the No. 4 Base Hospital which is being organised at King's College Hospital. Of the fifteen house officers, thirteen have received appointments in the Army or Navy Medical Services, and in order that the surgical work of the hospital may be kept at a high standard some of the visiting surgeons have been good enough to enter into residence as house surgeons. A great many offers of assistance have been received from old St. George's men to fill up the vacancies.

### ST. MARY'S HOSPITAL.

*(From our Special Correspondent.)*

At St. Mary's Hospital the resident medical officers volunteered practically *en masse* on the first day of the call; out of thirteen, we understand, only three remain to run the hospital. But as the out-patient department is closed, and several of the wards are being cleaned, the work has been materially reduced, and now assistance is forthcoming the hospital will be able to carry on quite satisfactorily. We hear that at least four of the late residents are already in Belgium. Of the honorary staff there are indeed few left behind for active work in the hospital, all the surgeons having taken up their posts in various base hospitals. Mr. Zachary Cope has remained at his post, and is, in fact, doing most of the surgical work in this institution.

### WESTMINSTER HOSPITAL.

*(From our Special Correspondent.)*

Beds to the number of 150 have been placed at the disposal of, and accepted by, the War Office. The following members of the medical staff have already left in various capacities for active service: The senior surgeon, Major C. Stonham, who is with the Yeomanry; the senior physician, Dr. Gossage, who, with Dr. Purves Stewart, physician to the hospital, is attached to No. 4 General Hospital, now established at King's College Hospital. Dr. Carmalt Jones, physician, has joined the Artists' contingent of the Territorials. The following surgeons have also left, and are likewise attached to No. 4 General Hospital:—Mr. W. G. Spencer, Mr. A. Tubby, Mr. W. Turner, and Mr. Arthur Evans, Mr. E. Rock Carling, out-patient surgeons. All the resident medical officers at Westminster Hospital but two have left, three for the Navy, who have already embarked from Liverpool, and four for the Army. Their names are: With the Navy: Dr. H. B. Carlill, medical registrar; Mr. G. J. C. Smyth, obstetric house physician. With the Army are: Mr. R. J. Clausen, house physician; Mr. Gwyn Davies, house physician; Mr. H. B. F. Dixon, house surgeon; Mr. B. Woodhouse, house surgeon; Mr. A. R. Hacker, house surgeon.

It is interesting to note that the vacant resident posts have been filled by volunteers, many of whom are not only old Westminster men, but also former residents. In fact, more offers to fill vacancies on the resident and honorary staff have been received than are required for the present. But the result is that the work of the hospital is not suffering, since the new residents are mostly senior men of very good qualifications who happen to be in this country and available at this time.

### UNIVERSITY COLLEGE HOSPITAL.

At the University College Hospital the whole of the resident staff have proceeded on active service with either the Army or Navy, and their work is now being done by old students of the hospital as volunteers. Twenty-four nurses have also gone, and others will follow. Half the consulting staff have been mobilised for service either with the Territorial Army or in connection with No. 3 Base Hospital. The authorities of the hospital have placed at the disposal of the Admiralty 100 beds for their use if required. Arrangements are being made by which the new Chemical Buildings at University College can in the case of necessity be fitted up and utilised as an annexe to the hospital. Special courses of instruction in dressing and nursing for the cadets of the Officers Training Corps have been instituted.

One of the greatest difficulties the hospitals will have to contend with is the increase in the cost of drugs and dressings, some of which in due course may become almost unobtainable.

